# Biochemical association of regulatory variant of KLF14 genotype in the pathogenesis of cardiodiabetic patients

**DOI:** 10.3389/fendo.2023.1176166

**Published:** 2023-06-07

**Authors:** Abdullah Salah Alanazi, Sumbal Rasheed, Kanwal Rehman, Tauqeer Hussain Mallhi, Muhammad Sajid Hamid Akash, Nasser Hadal Alotaibi, Abdulaziz Ibrahim Alzarea, Nida Tanveer, Yusra Habib Khan

**Affiliations:** ^1^ Department of Clinical Pharmacy, College of Pharmacy, Jouf University, Sakaka, Al-Jouf, Saudi Arabia; ^2^ Health Sciences Research Unit, Jouf University, Sakaka, Al-Jouf, Saudi Arabia; ^3^ Department of Pharmaceutical Chemistry, Government College University, Faisalabad, Pakistan; ^4^ Department of Pharmacy, The Women University, Multan, Pakistan; ^5^ Institute of Molecular Cardiology, University of Louisville, Louisville, KY, United States

**Keywords:** KLF14 rs4731702, single nucleotide polymorphism, Tetra-ARMS-PCR, dyslipidemia, genotypic analysis, diabetes mellitus, risk factors

## Abstract

**Background and purpose:**

The study focuses on examining the relationship between a single nucleotide polymorphism (SNP) in KLF14 rs4731702 and risk of type 2 diabetes mellitus (T2DM) and dyslipidemia in different ethnic populations. The purpose of this study was to evaluate the association between KLF14 rs4731702 and serum lipid profile and to determine the frequency distribution of KLF14 rs4731702 among T2DM and cardiometabolic patients.

**Methods:**

A total of 300 volunteers were recruited, consisting of three groups: 100 healthy individuals, 100 individuals diagnosed with T2DM, and 100 individuals diagnosed with cardiometabolic disorders. Biochemical analysis of blood samples was conducted to assess various biomarkers related to glycemic control and lipid profile. This involved measuring levels of glucose, triglyceride (TG), low-density lipoprotein cholesterol (LDL-C), high-density lipoprotein cholesterol (HDL-C), and ApoA1. Genotyping analysis was performed to investigate KLF14 rs4731702 polymorphism. The Tetra ARMS-PCR method was employed for genotyping analysis.

**Results:**

The results of biochemical profiling revealed a significant association between altered glycemic biomarkers and lipid profile in diseased patients compared to healthy participants. The frequencies of KLF14 rs4731702 alleles and genotypes were compared between the control group and T2DM group. A statistically significant difference was observed, indicating a potential association between KLF14 rs4731702 and T2DM. In the dominant inheritance model of KLF14 rs4731702 SNP, a statistically significant difference [odds ratio (95% confidence interval)] of 0.56 (0.34 –0.96) was found between the control and T2DM subjects. This suggests that the presence of certain genotypes influences the risk of T2DM. In T2DM patients, individuals carrying the C allele exhibited compromised insulin sensitivity, decreased HDL-C and ApoA1 levels, and increased serum glucose, TG, and LDL-C concentrations. Conversely, TT genotype carriers demonstrated increased levels of HDL-C and ApoA1, lower insulin resistance, serum glucose, LDL-C, and TG levels.

**Conclusion:**

The study’s findings indicate that dyslipidemia in T2DM patients is associated with reduced KLF14 functionality due to CC and CT genotypes, leading to insulin resistance and an increased risk of cardiovascular diseases. Additionally, risk of KLF14 rs4731702 polymorphism was found to increase with age and was more prevalent in female than in male individuals. These insights contribute to understanding genetic factors influencing the development and progression of T2DM and dyslipidemia in different ethnic populations.

## 1 Introduction

Type 2 diabetes mellitus (T2DM) is one of the chronic metabolic disorders. It is also known as non-insulin-dependent diabetes or adult-onset diabetes and is characterized by an imbalance in glucose levels in the body (1). According to the World Health Organization (WHO), approximately 415 million people globally have diabetes (2). Without proper medical care and monitoring, it can lead to other serious metabolic syndromes, including cardiovascular and kidney diseases (1). Insulin resistance is a prime symptom of T2DM, where cells fail to respond to insulin. Furthermore, if the progression of diabetes is not properly controlled, it disrupts the normal balance of lipids in the body, ultimately causing dyslipidemia (3). Several investigational studies have revealed that dyslipidemia increases the risk of cardiovascular diseases, particularly coronary artery heart diseases. Therefore, patients with dyslipidemia are more prone to developing coronary heart diseases (4).

DNA polymorphism is considered one of the main factors strongly associated with the pathogenesis of disease (5). Several genome-wide studies have revealed that numerous genes greatly contribute to different diseases. Approximately 200 genetic loci have been identified to be associated with diabetes mellitus (6). Therefore, exploring the genetic causes and risk factors is essential for the treatment and prevention of T2DM (7). KLF14, a master trans-regulator gene, belongs to the Kruppel-like family of transcription factors. It is a single axon imprinted gene located on chromosome 7q32.3, with a length of 1,059 bp, encoding 323 amino acids, and only the maternally transmitted allele is expressed (8). KLF14 is strongly associated with the expression of multiple metabolic traits, such as diabetes and obesity (9).

Genome-wide association studies (GWAS) have revealed that single nucleotide polymorphisms (SNPs) in the KLF14 gene are robustly associated with a multitude of metabolic pathologies, including T2DM, dyslipidemia, insulin resistance, coronary artery diseases, ischemic heart attack, and myocardial infarction (10–13). Moreover, human genetic studies have shown that variants near the KLF14 gene are strongly associated with T2DM, altered HDL-C and TG levels, and the risk of coronary artery disease (14). Although a strong association between the KLF14 gene polymorphism and both diabetes mellitus and cardiac problems has been found, the exact mechanisms by which it affects these conditions are still unknown. Some studies have suggested that the KLF14 gene polymorphism may interact with other genetic and environmental factors to increase the risk of both diabetes mellitus and cardiac problems. Due to its strong association with metabolic disorders, KLF14 is often referred to as the “conductor of the metabolic syndrome orchestra “ (15). KLF14 rs4731702 has a significant correlation with T2DM, HDL-C, and heart diseases. It is located approximately 14 kb upstream of the transcription-starting codon of the KLF14 gene (12, 13). In lipoprotein metabolism, KLF14 rs4731702 acts as an important regulator. Several studies have revealed that the maternally transmitted T allele of the rs4731702 SNP is associated with the upregulation of KLF14 gene expression in adipose tissues (16). The present study aims to detect the distribution of KLF14 rs4731702 SNP genotypes and evaluate the association of the SNP with serum lipid levels, serum glucose profile, ApoA1, Hb1Ac, total protein, hemoglobin, HOMA-IR levels, etc., in T2DM and cardiometabolic patients.

## 2 Materials and methods

### 2.1 Study design and study population

This case–control study aimed to determine the frequency of the KLF14 genotype in patients with type 2 diabetes mellitus (T2DM) and cardiometabolic disorders. The KLF14 rs4731702 polymorphism was assessed using Tetra-ARMS PCR. Blood samples were collected from two major hospitals in Faisalabad, namely, Allied Hospital, Faisalabad, and Social Security Hospital, Faisalabad. A total of 100 healthy controls, 100 diabetic individuals, and 100 diabetic patients with cardiac diseases, aged between 30 and 70 years, were included in the study. The protocol for this study received approval from the Ethical Review Committee of Government College University, Faisalabad, Pakistan (Ref. No. GCUF/ERC/33). Written informed consent was obtained from all enrolled patients, who were residents of Punjab, Pakistan. The recruitment of participants took place consecutively from November 2021 to March 2022.

### 2.2 Standards for inclusion and exclusion of study participants

Subjects between the ages of 30 and 70 years were included in this study. No participants under the age of 30 or over the age of 70 were enrolled. Patients with hepatic, renal, central nervous system (CNS) disorders, and endocrinal diseases were excluded from the study. Pregnant women and individuals using lipid-lowering drugs that could affect blood glucose metabolism and/or insulin sensitivity were not included. In summary, individuals with a medical history or any disorder, and those who used long-term lipid-lowering medication for comorbidities, were excluded from this study.

### 2.3 Blood sampling

Approximately 5 ml of blood was collected from each participant for biochemical analysis and genotyping testing. Out of this, approximately 2.5 ml of blood sample was collected in a gel clot activator vacutainer to obtain blood serum. The serum sample was obtained by centrifuging the sample at 12,000 rpm for 5–10 min at 4°C. The remaining 2.5 ml of blood was collected in EDTA tubes for DNA extraction and genotyping analysis. The extracted DNA samples and blood serum samples were aliquoted and stored at −20°C.

### 2.4 Anthropometric and clinical data

Vital signs including pulse, blood pressure, respiration rate, and temperature were assessed upon presentation. A traditional mercury sphygmomanometer was used to measure the systolic and diastolic pressures in millimeters of mercury (mmHg). The height and weight of each participant were measured to calculate the body mass index (BMI). BMI was calculated using the following formula:


(1) $$BMI=\frac{weight\;\left(kg\right)}{\left[height\;\left(m\right)\right]2}$$

BMI is commonly utilized to establish weight classification criteria according to clinical guidelines provided by the WHO. Individuals with a BMI<18.5 kg/m^2^ are classified as underweight, those with a BMI ranging from 18.5 to 24.9 kg/m^2^ are considered normal, individuals with a BMI between 25.0 and 29.9 kg/m^2^ are classified as overweight, and individuals with a BMI >30 are categorized as obese.

### 2.5 Biochemical analysis

The serum samples obtained from the collected blood were utilized to perform biochemical analysis of various parameters. Serum glucose, total cholesterol (TC), triglycerides (TGs), low-density lipoprotein cholesterol (LDL-C), high-density lipoprotein cholesterol (HDL-C), and serum total protein levels were determined using their respective assay kits on a biochemical analyzer (Microlab-300, ELITech Group, USA). Additionally, ApoA1, HbA1c, and serum insulin levels were measured using the ELISA kit method with an absorbance microplate reader (BioTek 800 TS absorbance reader, Agilent Technologies, USA).

### 2.6 Extraction of DNA

Genomic DNA was manually extracted from the collected blood specimens. The blood was pipetted into Eppendorf tubes and mixed with RBC lysis buffer. The mixture was then shaken well and centrifuged for 2 min at 7,000 rpm. The pellet was broken using a vortex mixer and rinsed with RBC lysis buffer. Next, nucleic acid lysis buffer, saturated NaCl (5 M), and chloroform were added to the Eppendorf tube. The mixture was mixed well and centrifuged for 2 min at 7,000 rpm. The supernatant was carefully transferred to a new Eppendorf tube and then centrifuged for 1 min at 12,000 rpm in the presence of cold ethanol. After discarding the supernatant, TE buffer was added to the pellet and vortexed. The Eppendorf tube containing the DNA for genotyping was stored at −20°C.

### 2.7 Purification and estimation of DNA

After DNA extraction, qualitative and quantitative analyses of the extracted DNA were carried out using different techniques. The quantitative analysis was performed using the NanoDrop method, while the qualitative analysis was conducted through gel electrophoresis. The estimated DNA quantity was determined by measuring the 260/280 absorbance ratio using the NanoDrop method.

### 2.8 Primer designing and KLF14 rs4731702 genotyping

**Figure 1:**
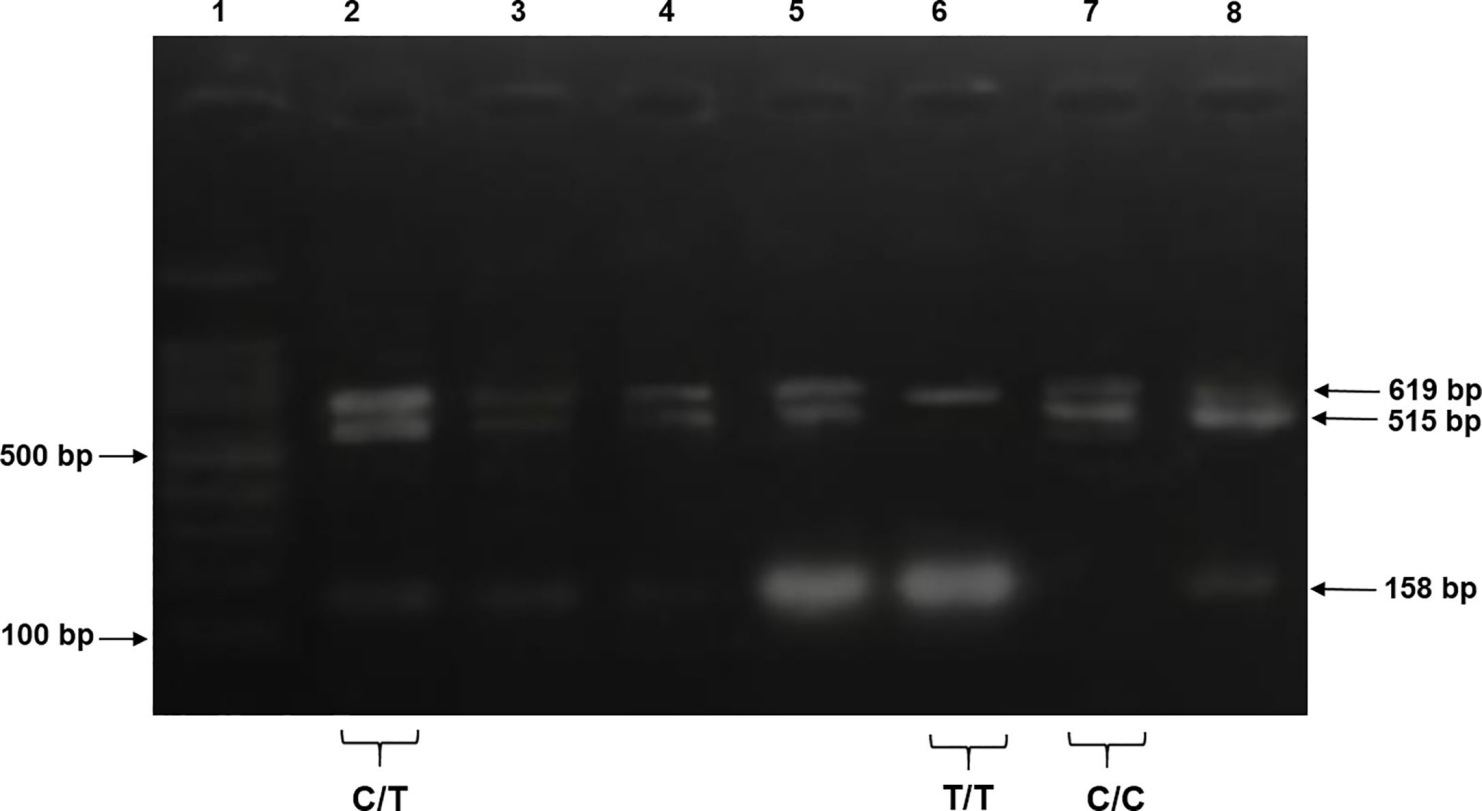
The products were electrophoresed on 2% agarose and directly visualized with ethidium bromide under UV light. Lane 1: 100 bp DNA ladder, lanes 2–5 and 8 indicate samples with heterozygous genotype CT CC (619 + 515 + 158 bp), lane 6 indicates homozygous genotype TT (619 + 158 bp), and lane 7 indicates a sample of homozygous genotype CC (619 + 515 bp).

**Figure 2:**
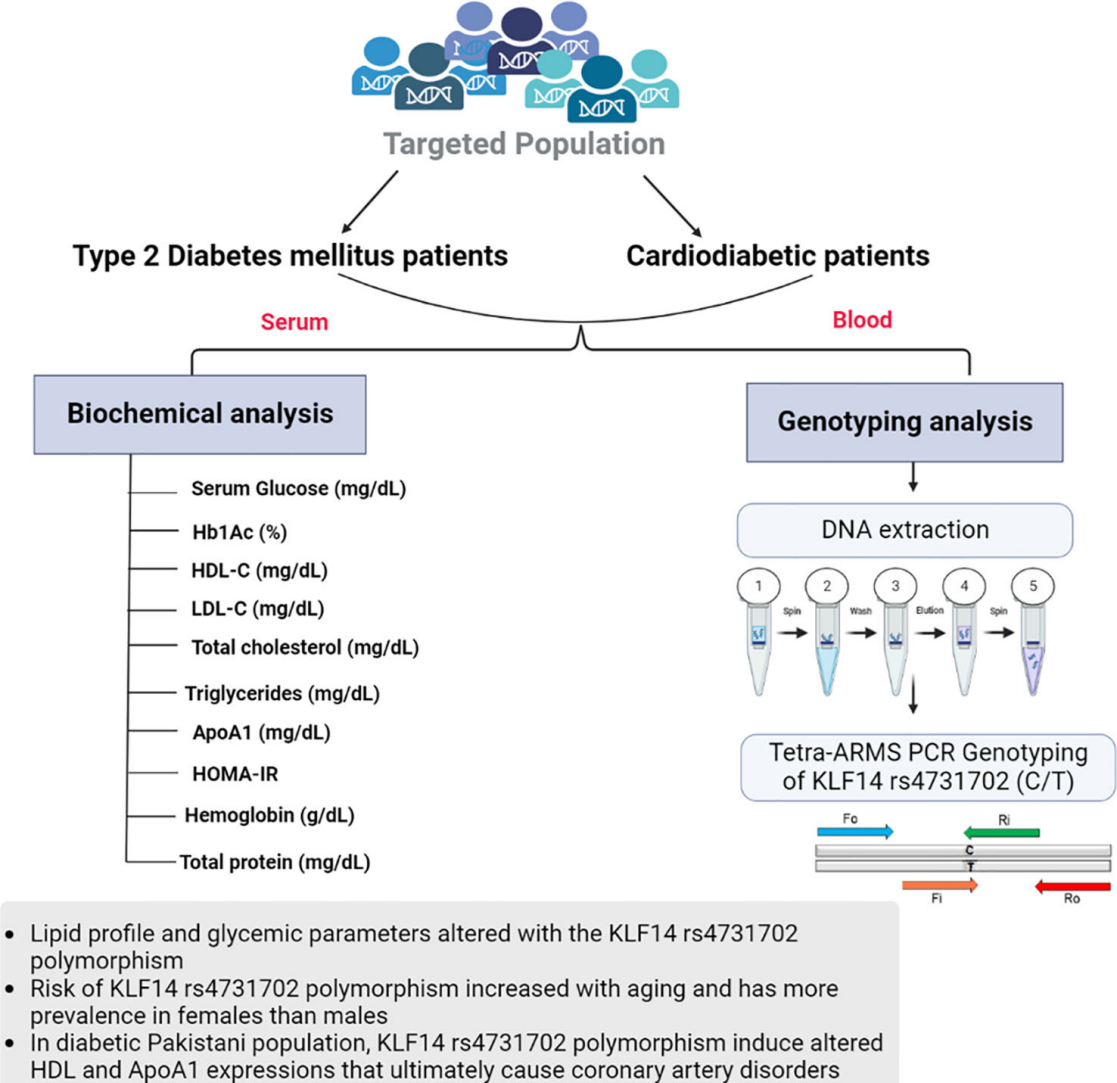
Graphical representation of biochemical analysis and KLF14 rs4731702 (C/T) genotyping analysis.

For tetra-ARMS PCR, two inner and two outer primers were designed and synthesized using the Primer-BLAST software and ordered from Custom DNA Oligos-Eurofins Genomics. The primer sequences are listed in **Table 1**. The PCR reaction was performed in a total volume of 20 μl, consisting of 10 μM 2× PCR Taq Plus Master Mix with dye (BioShop, Canada Inc.), 220 ng of DNA template, 1 μl of inner primer for the C allele at 0.2 pmol, 1 μl of inner primer for the T allele at 0.4 pmol, 1 μl of reverse outer primer at 0.2 pmol, 1 μl of forward outer primer at 0.4 pmol, and 8 μl of PCR water. The tetra-ARMS PCR was conducted to determine the genotypic polymorphism of the KLF14 gene rs4731702. The PCR process was performed using the “Thermocycler Master Cycler Gradient.” The PCR reaction conditions were optimized for the amplification of the target DNA segment, and the following temperature profile was used: initial denaturation at 95°C for 3 min, followed by 35 cycles of denaturation at 95°C for 1 min, annealing at 65°C for 1 min, extension at 72°C for 1 min, and a final extension at 72°C for 5 min (**Table 2**). Agarose gel electrophoresis (2%) stained with ethidium bromide was performed to separate the resulting DNA amplicons. The DNA bands were visualized under UV light transillumination and captured in photographs (17), as shown in **Figure 1**. A schematic representation of this case–control study is depicted in **Figure 2**.

**Table 1:** For the Tetra ARMS PCR, primers’ sequences of KLF14 gene.

Gene primers	Primer sequence
Forward inner primer	AAAAAACAGCATTATTTCCCACACAAAC
Forward outer primer	CCCAAGGCATCTATCCAAAA
Reverse inner primer	TATCTTTTTGGTGCTAAATGGAACGGA
Reverse outer primer	CCGTTGAACTGTGTTTGCAC

**Table 2:** Tetra ARMS PCR profile for the KLF14 rs4731702 (C/T).

Reaction condition	Temperature	Time
Initial denaturation	95	3 min
Denaturation	95	1 min
Annealing	65	1 min
Extension	72	1 min
No. of cycles	30	–
Final extension	72	4 min

### 2.9 Statistical analysis

For the statistical analysis, GraphPad Prism 5 and Minitab software were utilized. The significant differences among the study groups were analyzed using one-way ANOVA followed by Tukey’s *post-hoc* test. Fisher’s exact test was employed to calculate the significant differences in genotypic frequencies of KLF14 rs4731702 among the study groups. The software SNP Stats was used for the genotyping analysis, which determined the distribution of allelic and genotypic frequencies.

## 3 Results

### 3.1 Biochemical analysis

Biochemical parameters were analyzed in the study subjects, as shown in **Table 3**. Serum levels of Apo-A1, HDL-C, LDL-C, TC, TGs, glucose, Hb1Ac, HOMA-IR, and total protein were found to be significantly different (p< 0.05) between the diabetic and cardiodiabetic patients when compared to the normal controls. Additionally, significant differences were observed among the diabetic and cardiodiabetic patients themselves (**Table 3**).

**Table 3:** Biochemical parameters of the study participants.

Biochemical parameters	Normal participants (n=100)	Diabetic patients (n=100)	Cardiodiabetic patients (n=100)
Glucose (mg/dl)	115.5 ± 5.319	155.5 ± 8.324*	207.7 ± 15.33*^
Hb1Ac (%)	4.864 ± 0.3448	6.187 ± 0.4141*	8.841 ± 0.5914*^
HOMA-IR	0.5390 ± 0.1197	1.892 ± 0.3437*	1.860 ± 0.3130*^
TC (mg/dl)	163.6 ± 13.49	226.7 ± 7.687*	257.3 ± 8.245*^
LDL-C (mg/dl)	116.6 ± 7.886	146.4 ± 7.004*	178.6 ± 9.844*^
HDL-C (mg/dl)	66.50 ± 5.526	43.88 ± 5.566*	30.88 ± 6.602*^
TG (mg/dl)	135 ± 8.079	175.1 ± 10.10*	329.8 ± 62.08*^
Apo A1 (mg/dl)	138.6 ± 13.82	87.37 ± 26.88*	66.04 ± 8.159*^
Total protein (mg/dl)	7.546 ± 0.4738	7.973 ± 0.8883*	8.354 ± 0.3119*^
Hemoglobin (g/dl)	13.67 ± 1.158	10.34 ± 1.126*	10.38 ± 1.374*

This analysis was caried out by GraphPad prism (version 5.01) using one-way ANOVA test

*vs. normal participants, ^vs. diabetic patients.

### 3.2 Participant characteristics

Among diabetic and cardiometabolic patients, the percentage of smokers was 33% and 23%, respectively. In the diabetic group, women accounted for 36%, while men accounted for 29%. In the cardiodiabetic group, women accounted for 51%, while men accounted for 44%. Furthermore, the prevalence of a family history of diabetes was 43% in the diabetic group and 49% in the cardiodiabetic group (**Table 4**).

**Table 4:** Sociodemographic and anthropometric parameters of the study participants.

Parameter	Total subjects (n = 200)		
	Healthy individuals (n = 100)	T2DM patients (n = 200)	
		Diabetic	Cardiodiabetic
Participants	100 (33%)	100 (33%)	100 (33%)
Gender wise distribution (Sex)			
Females	70 (37%)	67 (36%)	51 (27%)
Males	30 (27%)	33 (29%)	49 (44%)
Smoking status			
Smoker	12 (12%)	33 (33%)	25 (25%)
Non-smoker	88 (88%)	67 (67%)	75 (75%)
Age (years)			
Age	39.30 ± 11.99	44.59 ± 13.76	48.30 ± 12.86
Blood pressure (mmHg)			
Systolic BP	121.7 ± 5.477	120.9 ± 4.852	148.1 ± 15.55
Diastolic BP	76.89 ± 2.961	77.56 ± 2.702	85.46± 5.217
Body fat status			
BMI (kg/m^2^)	21.87 ± 2.209	26.05 ± 3.085	28.81 ± 2.520
Family history of diabetes			
Yes	22	43	49
No	78	57	51

### 3.3 Genotyping and KLF14 rs47331702 polymorphism

PCR products of KLF14 rs4731702 were generated using Tetra-ARMS PCR to determine the genotypes (CT, TT, and CC). The outer primer produced a 619-bp band, serving as the amplicon for the outer primers. The C allele was identified by a band size of 515 bp, resulting from the combination of an outer primer and the inner primer. Conversely, the T allele was represented by a 158-bp band generated by another set of inner and outer primers (**Figure 1**).

### 3.4 Genotype and allele frequencies of KLF14 gene polymorphisms in diseased patients and controls according to the Hardy–Weinberg equation

**Table 5** illustrates the allelic and genotypic frequencies of the KLF14 gene polymorphisms among disease patients and controls, calculated according to the Hardy–Weinberg equation. The statistical analysis showed that neither of these populations theoretically followed the Hardy–Weinberg equation, as indicated by a significant (p< 0.05) difference revealed by the Chi-test between the diseased patients and controls. These results demonstrate that the allelic and genotypic frequencies of KLF14 rs4731702 vary across different generations.

**Table 5:** Genotyping and allelic frequency distribution among control and diseased groups.

Status	Genotype/Allele KLF14 rs4731702					*Hardy–Weinberg Equilibrium* *(p*-value)	*95% confidence interval*
	TT	CT	CC	C	T		
Normal control (n=100)	23	49	28	105	95	0.84	12.87–107.1
Disease cases (n=200)	34	85	81	247	153	0.18	17.31–222.7
Chi- square (X^2^)	0.049						

The p-value was calculated by SNPstats software (http://bioinfo.iconcologia.net/SNPstats) by using Fisher’s exact test and Hardy–Weinberg equillibrium analysis.

### 3.5 Genotype and allele frequencies of KLF14 gene polymorphisms in T2DM patients and cardiodiabetic subjects according to Hardy–Weinberg equation

**Table 6** presents the allelic and genotypic frequencies of KLF14 rs4731702 (C/T) among the diseased groups: the diabetic group and cardiodiabetic group. The statistical analysis indicated that both of these populations theoretically follow the Hardy–Weinberg equation, as the Chi-test revealed a non-significant (p > 0.05) difference between diabetic and cardiodiabetic patients. These results demonstrate that the allelic and genotypic frequencies of KLF14 rs4731702 remain consistent across different generations.

**Table 6:** Genotyping and allelic frequency distribution among diabetic and cardiodiabetic group*s*.

Status	Genotype/allele KLF14 rs4731702					*Hardy–Weinberg equilibrium (p*-value)	*95% confidence interval*	
	TT	CT	CC	C	T			
T2DM(n=100)	19	45	35	115	83	0.53	10.98–107.8	
Cardiodiabetic (n=200)	15	40	46	132	70	0.2	5.408–115.8	
Chi Square (X^2^)	0.3457						

The p-value was calculated by SNPstats software (http://bioinfo.iconcologia.net/SNPstats) by using Fisher’s exact test and Hardy-Weinberg equillibrium analysis.

### 3.6 Inheritance model for KLF14 rs4731702 among controls and diseased

The association between KLF14 genotype and T2DM was analyzed using all possible genetic inheritance models. The findings revealed a significant difference in the heterozygous genotype (C/T) under the dominance model [OR (95% CI) 0.56 (0.34 –0.96), p< 0.05]. However, no significant differences were observed under the codominance [OR (95% CI) 0.60 (0.34 –1.04), p > 0.05], overdominance [OR (95% CI) 0.77 (0.48 –1.25), p > 0.05], and recessive models [OR (95% CI) 0.69 (0.38 –1.24), p > 0.05]. Therefore, the statistical analysis demonstrated a significant difference between the healthy and diseased groups specifically under the dominance model (**Table 7**).

**Table 7:** Inheritance models for the genotyping and allelic frequencies of KLF14 gene among control and diseased groups.

Model	Genotype	Control	Diseased	OR (95% CI)	p-value
Codominant	C/C	28 (28%)	81 (40.5%)	1.00	0.089
	C/T	49 (49%)	85 (42.5%)	0.60 (0.34 –1.04)	
	T/T	23 (23%)	34 (17%)	0.51 (0.26–1.01)	
Dominant	C/C	28 (28%)	81 (40.5%)	1.00	0.032
	C/T– T/T	72 (72%)	119 (59.5%)	0.56 (0.34– 0.96)	
Recessive	C/C– C/T	77 (77%)	166 (83%)	1.00	0.22
	T/T	23 (23%)	34 (17%)	0.69 (0.38– 1.24)	
Overdominant	C/C– T/T	51 (51%)	115 (57.5%)	1.00	0.95
	C/T	49 (49%)	85 (42.5%)	0.77 (0.48– 1.25)	
Log -additive	–	–	–	0.70 (0.50– 0.98)	0.037

The p-valuewas calculated by SNPstats software (http://bioinfo.iconcologia.net/SNPstats) by using Fisher’s exact test and Hardy–Weinberg equillibrium analysis.

### 3.7 Inheritance model for KLF14 rs4731702 among T2DM and cardiodiabetic groups

The association of KLF14 genotype among T2DM and cardiodiabetic groups was analyzed using all possible genetic inheritance models. The findings revealed no significant differences under the codominance [OR (95% CI) 0.69 (0.37 –1.28), p > 0.05], dominance [OR (95% CI) 0.66 (0.38 –1.17), p > 0.05], recessive [OR (95% CI) 0.73 (0.35 –1.52), p > 0.05], and overdominance models [OR (95% CI) 0.80 (0.46 –1.41), p > 0.05]. Therefore, the statistical analysis showed no significant difference between the diabetic and cardiodiabetic groups under all inheritance models (**Table 8**).

**Table 8:** Inheritance models for the genotyping and allelic frequencies of KLF14 gene among diabetic and cardiodiabetic groups.

Model	Genotype	T2DM	Cardiodiabetic	OR (95% CI)	p-value
Codominant	C/C	35 (35.7%)	46 (45.5%)	1.00	0.35
	C/T	44 (44.9%)	40 (39.6%)	0.69 (0.37 –1.28)	
	T/T	19 (19.4%)	15 (14.8%)	0.60 (0.27–1.35)	
Dominant	C/C	35 (35.7%)	46 (45.5%)	1.00	0.16
	C/T –T/T	63 (64.3%)	55 (54.5%)	0.66 (0.38 –1.17)	
Recessive	C/C –C/T	79 (80.6%)	86 (85.2%)	1.00	0.4
	T/T	19 (19.4%)	15 (14.8%)	0.73 (0.35 –1.52)	
Overdominant	C/C –T/T	54 (55.1%)	61 (60.4%)	1.00	0.45
	C/T	44 (44.9%)	40 (39.6%)	0.80 (0.46 –1.41)	
Log -additive	–	–	–	0.76 (0.51– 1.12)	0.16

The p-value was calculated by SNPstats software (http://bioinfo.iconcologia.net/SNPstats) by using Fisher’s exact test and Hardy–Weinberg equillibrium analysis.

### 3.8 Allelic/genotyping frequency of diabetic patients according to their gender, age, body weight, and smoking status

The allelic frequency of KLF14 was evaluated in the diseased population (n = 200) with respect to gender, age, and smoking history. Among diabetic patients, no statistically significant difference in KLF14 genotype frequency was found between smokers and non-smokers. Additionally, in the diseased groups, 54% of patients were overweight, and 24.5% were obese. The frequency of the CT genotype of the KLF14 gene was higher in obese subjects (65%) compared to overweight and healthy individuals (**Table 9**).

**Table 9:** Distribution of KLF14 genotypes and allelic frequencies in diseased population, in accordance with age, gender, obesity, and smoking.

Parameters	T2DM patients with and without cardiac complications (n = 200)			
	No. of T2DM patients	TT	CT	CC
Female	118	16 (14%)	54 (46%)	48 (41%)
Male	82	18 (22%)	31 (38%)	33 (40%)
Healthy (18–25 kg/m^2^)	43	9 (20%)	14 (32%)	20 (46%)
Overweight (25–30 kg/m^2^)	108	21 (19%)	39 (36%)	48 (44%)
Obese (>30 kg/m^2^)	49	4 (8%)	32 (65%)	13 (27%)
Smoker	58	14 (24%)	23 (40%)	21 (36%)
Non-smoker	142	20 (14%)	62 (44%)	60 (42%)
Elderly (> 40)	111	19 (17%)	49 (44%)	43 (39%)
Young (< 40 years)	89	33 (37%)	29 (33%)	27 (30%)

The genotypic frequency of KLF14 rs4731702 showed that the CT and CC genotypes were significantly higher (p< 0.05) in women (46% and 41%, respectively) compared to men (38% and 40%, respectively). Furthermore, the frequency of the CT genotype was higher in elderly diabetic patients (44%) compared to young diabetic patients. In conclusion, KLF14 C carrier genotypes may increase the risk of diabetes pathogenesis. The genotypic results also indicated that the chances of KLF14 rs4731702 (C/T) polymorphism increase with aging. Obesity showed no association with the KLF14 rs4731702 polymorphism, and similar results were found regarding smoking, indicating no significant association between smoking and the KLF14 rs4731702 polymorphism. However, a significant risk association was found between diabetes mellitus and the KLF14 rs4731702 polymorphism in women compared to men (**Table 9**).

### 3.9 Association between KLF14 rs4731702 polymorphism and tested biochemical, clinical, and anthropometric parameters

The clinical and biochemical parameters were analyzed in the study subjects in relation to the KLF14 genotypic polymorphism. The genotypic frequencies of KLF14 rs4731702 in the study sample are presented in **Table 10**. KLF14 genotypes containing the C allele were associated with significantly lower levels of HDL-C and Apo A1 in the diseased subjects (p< 0.05). Among T2DM patients, individuals with C allele carriers (CC and CT) showed a higher prevalence of altered serum lipid profiles compared to those with the TT genotype. KLF14 C-carrier genotypes had a significant impact on insulin resistance compared to the TT genotype. However, there was no statistically significant association observed between KLF14 rs4731702 SNP and biochemical parameters, including Hb1Ac and total protein levels, in the study subjects. Moreover, the prevalence of C carrier alleles was higher in women than in men within the study groups. Nevertheless, no statistically significant associations were found between KLF14 rs4731702 (C/T) polymorphism and clinical or anthropometric characteristics in the study subjects (**Table 10**).

**Table 10:** Association between KLF14 rs4731702 polymorphism and tested biochemical, clinical, and anthropometric parameters.

Parameters	Control				Diabetic				Cardiodiabetic			
	CC	CT	TT	P value	CC	CT	TT	P value	CC	CT	TT	P value
HDL-C (mg/dl)	59.86 ± 2.4	67.31 ± 3.0	72.87 ± 3.2	<0.05	40.69 ± 4.5	44 ± 4.46	49.20 ± 5.4	<0.05	25.59 ± 4.4	33.90 ± 3.87	39.64 ± 3.2	<0.05
LDL-C (mg/dl)	126.5 ± 2.36	116.0 ± 2.45	105.6 ± 2.67	<0.05	154.2 ± 2.2	144.2 ± 2.61	137.5 ± 4.69	<0.05	186.1 ± 8.05	175.2 ± 3.64	163.8 ± 1.67	<0.05
ApoA1 (mg/dl)	123.5 ± 6.22	139.4 ± 9.56	155.3 ± 5.21	<0.05	69.37 ± 7.5	79.73 ± 9.64	136.1 ± 14.76	<0.05	59.02 ± 4.42	68.83 ± 3.92	76.14 ± 5.31	<0.05
TGs (mg/dl)	134.4 ± 6.5	135.0 ± 8.9	135.7 ± 8.0	>0.05	235.3 ± 2.5	225.0 ± 2.5	215.5 ± 2.4	<0.05	380.1 ± 43.5	301.1 ± 33	246.2 ± 21.9	<0.05
TC (mg/dl)	178.6 ± 5.9	163.7 ± 4.4	145.0 ± 9.4	<0.05	235.3 ± 2.5	225.0 ± 2.5	215.5 ± 2.3	<0.05	264.6 ± 4.0	253.6 ± 3.01	244.0 ± 3.2	<0.05
Hb1Ac (%)	4.829 ± 0.3	4.876 ± 0.3	4.917 ± 0.3	>0.05	6.183 ± 0.3	6.158 ± 0.4	6.185 ± 0.3	>0.05	8.926 ± 0.6	8.815 ± 0.54	8.779 ± 0.5	>0.05
HOMA-IR	0.53 ± 0.1	0.55 ± 0.12	0.531 ± 0.1	>0.05	1.823 ± 0.3	2.069 ± 0.3	1.615 ± 0.2	<0.05	1.728 ± 0.2	2.050 ± 0.34	1.750 ± 0.3	<0.05
Fasting blood glucose (mg/dl)	114.5 ± 5.4	115.6 ± 5.0	116.4 ± 5.6	>0.05	164.0 ± 5.3	169.9 ± 3.1	154.5 ± 5.2	<0.05	166.5 ± 5.1	170.4 ± 2.22	157.3 ± 4.3	<0.05
Hemoglobin (g/dl)	13.95 ± 1	13.52 ± 1.1	13.66 ± 1.3	<0.05	10.22 ± 1	10.36 ± 1.2	10.52 ± 1.2	<0.05	10.25 ± 1.3	10.57 ± 1.5	10.3 ± 1.3	<0.05
Total protein (mg/dl)	7.59 ± 0.4	7.616 ± 0.4	7.339 ± 0.6	>0.05	7.951 ± 1.1	8.071 ± 0.2	8.100 ± 0.2	>0.05	8.350 ± 0.3	8.38 ± 0.29	8.28 ± 0.4	>0.05
Systolic BP	121.4 ± 6.3	121.7 ± 4.8	122.3 ± 5.7	>0.05	120.8 ± 4.26	121.7 ± 9.8	119.2 ± 5.37	>0.05	146.5 ± 15.14	149.3 ± 15.7	150.2 ± 16.93	>0.05
Diastolic BP	76.39 ± 3.31	76.55 ± 2.96	78.22 ± 2.08	>0.05	77.03 ± 2.8	77.69 ± 2.72.26	78.20±	>0.05	84.33 ± 5.4	86.80 ± 5.8	85.36 ± 4.12	>0.05
Female	16%	38%	16%	–	27%	32%	8%	–	21%	22%	8%	–
Male	12%	11%	7%	–	8%	13%	12%	–	25%	18%	6%	–
Age (<40)	15	32	18	–	21	22	14	–	22	19	2	–
Age (>40)	13	17	5	–	14	23	6	–	24	21	12	–
Smokers	3	7	2	–	9	13	11	–	12	10	3	–
Non-smokers	25	42	21	–	26	32	9	–	34	30	11	–
Obese	0	0	0	–	4	10	1	–	10	22	3	–
Non-obese	28	49	23	–	31	35	19	–	36	18	11	–

The p-value was calculated by GraphPad prism (version 5.01) by using one-way ANOVA and Tukey*’*s multiple comparison test

## 4 Discussion

T2DM is a prevalent and concerning health issue with a rising incidence rate worldwide (18). Diabetes is associated with various complications, including insulin resistance, glucose intolerance, and cardiac diseases, among others. Previously, diabetes was predominantly prevalent in urban areas, but due to urbanization, changes in lifestyle, modified dietary habits, and reduced physical activity, its prevalence has increased in underdeveloped countries like Pakistan (19). Pakistan, with a population of 207.7 million and a geographical area of 796,095 km^2^, ranks as the sixth most populated and the 36th largest country globally (20).

Both diabetic patients with and without cardiac complications have lower levels of HDL-C compared to healthy individuals. Triglyceride, low-density lipoprotein (LDL-C), and total cholesterol concentrations were found to be higher in T2DM and cardiodiabetic patients than in the control group. Furthermore, these lipid levels were significantly elevated in cardiodiabetic patients compared to those with T2DM alone. Consistent with these findings, our data indicate that compared to the control participants, both T2DM and cardiodiabetic patients exhibited higher levels of BMI, fasting blood glucose, and HbA1c, and lower levels of ApoA1. Diabetic patients also showed insulin resistance, which exacerbates the disease and increases the risk of complications, particularly coronary heart diseases. Consequently, the altered lipid parameters, including HDL-C, triglycerides, LDL-C, total cholesterol, and ApoA1, contribute to the pathogenesis of the disease. A schematic representation of this case–control study is depicted in **Figure 2**.

Additionally, there is evidence that dyslipidemia can cause insulin resistance, leading to hyperinsulinemia, which is a key indicator of developing T2DM. Implementing strategies to prevent and control the development of hyperinsulinemia can be a protective approach in reducing the risk of T2DM and its associated complications, including coronary artery disorders and atherosclerosis (21, 22). Insulin resistance occurs when body cells, particularly hepatocytes, adipocytes, and muscle cells, become resistant to insulin, resulting in impaired insulin sensitivity. Under certain conditions, these cells fail to respond adequately to insulin, leading to increased insulin secretion by pancreatic β-cells to maintain normal regulation of glucose metabolism and blood glucose levels. This condition is known as hyperinsulinemia. However, if the cells become excessively resistant, it leads to hyperglycemia, which gradually progresses to prediabetes and type 2 diabetes mellitus. Insulin resistance in individuals with type 2 diabetes mellitus has been linked to various health issues, including cardiovascular diseases, obesity, metabolic disorders, fatty liver disease, and polycystic ovarian syndrome.

Furthermore, the serum levels of hemoglobin and total protein also showed variations in the diabetic and cardiodiabetic groups compared to the control individuals. Amnesia, which is common in diabetic patients, contributes to the severity and progression of diabetes. Previous studies have indicated that low hemoglobin concentration is associated with cardiac diseases (23). Amnesia serves as a crucial marker for cardiovascular diseases and is considered a risk factor for severe coronary artery diseases (24). Total protein levels indicate the presence of proteinuria, and individuals with diabetes and heart disorders are at a higher risk of developing proteinuria. Diabetic proteinuria has been associated with the severity of diabetes, nephropathy, obesity, and elevated blood pressure (25). Proteinuria has a strong correlation with cardiovascular diseases (26).

In this research, the concentration of serum HDL-C was found to be significantly reduced in cardiodiabetic patients compared to diabetic and control participants. However, diabetic patients exhibited compromised levels of HDL-C compared to the control group. The main focus of this study was on the serum levels of HDL-C and ApoA1, which are key biochemical parameters. HDL-C, also known as high-density lipoprotein, plays a crucial role in cholesterol transport from peripheral tissues to the liver through a mechanism called reverse cholesterol transport (27). Increased levels of serum HDL-C are considered cardio-protective in the human body. HDL-C is a beneficial lipoprotein that not only regulates cholesterol transport but also possesses antioxidant, anti-thrombotic, and anti-inflammatory properties. Therefore, a decrease in serum HDL-C levels has been associated with cardiac complications such as atherosclerosis and myocardial infarction (28).

Apolipoprotein A1 (ApoA1), primarily secreted from the liver and kidney, is the major component of HDL-C and plays a crucial role in regulating reverse cholesterol transport. Abnormal or altered expression of ApoA1 and HDL-C can lead to hyperlipidemia, increasing the risk of atherosclerotic and other cardiac complications (29). Typically, cardiac patients exhibit reduced levels of serum ApoA1, which negatively affects their heart health. KLF14, a member of the KLF family, is a trans-regulatory gene that regulates various mechanisms, including gluconeogenesis. Studies have reported that overexpression of the KLF14 gene in hepatocytes restricts the inhibition of glucose uptake triggered by high levels of insulin and glucose (30). The KLF14 gene also plays a role in regulating the expression of ApoA1 protein and knockdown of the KLF14 gene results in ApoA1 deficiency (31). A case–control study investigating the association between serum lipid profiles and KLF14 rs4731702 revealed that in the Han population, carriers of the C allele had decreased levels of ApoA1 and HDL-C compared to carriers of the T allele (32).

In this research study, an altered lipid profile has been observed in patients with T2DM, potentially attributed to a decrease in KLF14 activity in these individuals due to dyslipidemia. Another possibility for the altered KLF14 expression is insulin resistance, which downregulates the normal expression of KLF14. The allelic and genotypic frequencies of KLF14 rs4731702 in the Pakistani population are currently unknown. There is no existing research indicating any ethnic relationship between the KLF14 rs4731702 genetic polymorphism and the Pakistani population. This gene has been studied in various ethnic populations, including European and Chinese populations. The minor T allele frequency has been reported as 36.7% in Chinese, 30.0% in Japanese, 23.3% in European, 56.1% in Icelander, and 30.0% in Yoruba populations (16). However, the minor allelic frequency of the T allele was investigated in patients with atherosclerotic cardiovascular disease in the Taizhou and Beijing Chinese populations. The results showed that the T allele frequency was lower in myocardial infarction and ischemic stroke patients compared to healthy participants (11). In this study, we also examined the association of age, sex, and smoking with KLF14 gene polymorphism, as ethnic diversity and environmental factors have been identified as strong factors for DNA mutations and genetic polymorphisms.

KLF14 plays a key role in the regulation of lipid metabolism as a master trans-regulatory gene that controls the normal expression of multiple genes associated with metabolic phenotypes in adipocytes (33). GWAS studies have shown that the T allele of KLF14 rs4731702 is associated with increased serum levels of HDL-C, which in turn enhances heart health and acts as a protective factor against cardiovascular diseases and type 2 diabetes mellitus. According to the Diabetes Genetics Replication and Meta-analysis (DIAGRAM) organization, the C allele of KLF14 rs4731702 is associated with an increased risk of type 2 diabetes mellitus (11, 12, 16).

Several studies have provided evidence that the minor T allele of KLF14 rs4741702 is maternally transmitted and linked to upregulation of the KLF14 gene in adipose tissues, indicating the presence of cis-expression quantitative trait loci (cis-eQTL) (16). An investigational study was conducted to determine the cis-acting eQTL of the KLF14 gene in female twins of European descent. The findings revealed a trans-regulatory association between the KLF14 gene and 10 genes that have been implicated in the regulation of several metabolic disorders, including dyslipidemia, insulin resistance, and obesity. Additionally, they found that 5 out of these 10 genes are located near SNPs that were significantly associated with key metabolic syndrome traits at a genome-wide significance level (33). According to an investigational study, the T allele of KLF14 rs4731702 has a significant association with elevated levels of serum HDL-C, which confers protective effects against cardiac illnesses and type 2 diabetes mellitus (11).

Interestingly, previous studies have shown that the risk allele for T2DM in KLF14 rs4731702 is the C allele, which significantly downregulates KLF14 expression in adipose tissues (16). Therefore, it can be inferred that patients with T2DM and the CC genotype have altered KLF14 expression, resulting in lower levels of HDL-C compared to T allele carriers. The risk C allele of KLF14 rs4731702 is associated with insulin resistance, which increases the risk of T2DM (12). Consistent with our present research study, our findings showed that patients with T2DM, both with and without cardiac dysfunction, and the CC genotype have reduced serum levels of HDL-C, ApoA1, and insulin sensitivity. Furthermore, the prevalence of KLF14 rs4731702 (C/T) polymorphism increased with age, and the genotyping frequency was higher in women than in men. However, no association was found between smoking and KLF14 polymorphism.

In line with our findings, T2DM patients with the CC genotype had higher levels of total cholesterol and lower levels of HDL-C, increasing the risk of insulin resistance. Therefore, these diabetic patients are at a higher risk of developing cardiac disorders. It can be concluded that C-carrier diabetic patients are associated with a risk of cardiovascular disorders. However, a comprehensive study is needed to fully understand the mechanism of KLF14 rs4731702 SNP in lipid metabolism. Limitations of this study include a small sample size, limited time, comorbidities, and limited resources.

## 5 Recommendations and future aspects

In developing countries, including Pakistan, conducting allele-specific genotyping studies to identify DNA mutations at the single nucleotide level is rare. This study successfully identified a single allelic-level genetic mutation in the KLF14 gene among the diabetic population. This mutation indicates that a specific polymorphism in patients with T2DM can lead to coronary artery diseases, including atherosclerosis and myocardial infarction. The study falls within the realm of pharmacogenetics, which aims to elucidate disease pathogenesis and guide the development of targeted therapeutic agents (precision medication). In the current era, personalized medication techniques have gained worldwide attention due to their reduced adverse effects, enhanced therapeutic efficacy, and lower disease recurrence rates. This study represents a valuable contribution to the field of precision medication. By associating diseases with genetic polymorphisms, it becomes possible to minimize medication errors and treatment costs. Given that diabetic patients with C allele carriers are predisposed to dyslipidemia, there is a likelihood that these individuals may experience atherosclerotic heart diseases in the near future. Therefore, it is advisable to recommend precision medication for the treatment of dyslipidemia, aiming to prevent cardiac complications in diabetic patients. However, it is essential to emphasize the need for conducting this study on a larger sample size in order to achieve statistical validity and ensure the reliability of data analysis.

## 6 Conclusions

Our study revealed that both diabetic and cardiac diabetic patients have altered lipid profiles, ApoA1 levels, serum glucose, HOMA-IR, and total protein levels when compared to the control participants group. The present study also evidenced a substantial association between serum lipid profile and the KLF14 rs4731702 in disease patients. Diabetic patients with C allele frequency had greater susceptibility to have an altered lipid profile and ApoA1 levels and insulin resistance than T allele carriers. Thus, allele C carriers, more specifically C/C genotypic diabetic patients, are at higher risk of developing cardiac diseases. Moreover, KLF14 rs4731702 (C/T) polymorphism was found to be more prevalent in women than men and increased with age, although smoking did not correlate with KLF14 rs4731702 polymorphism. Moreover, the study underscores the potential of pharmacogenetics in elucidating disease pathogenesis and guiding the development of targeted therapeutic agents. The identification of genetic mutations at the allelic level provides valuable insights into the molecular mechanisms underlying disease progression and highlights the significance of precision medicine in optimizing treatment outcomes. Overall, this study contributes to the growing body of knowledge on the genetic determinants of dyslipidemia and cardiovascular diseases in patients with T2DM, paving the way for future advancements in precision medicine and personalized treatment strategies for individuals at risk of cardiovascular complications.

## Data availability statement

The original contributions presented in the study are included in the article/supplementary material. Further inquiries can be directed to the corresponding authors.

## Ethics statement

The studies involving human participants were reviewed and approved by the Ethical Review Committee of Government College University, Faisalabad, Pakistan (Ref. No. GCUF/ERC/33). The patients/participants provided their written informed consent to participate in this study.

## Author contributions

Conceptualization: MSHA and KR. Data curation: SR, KR, and NA. Formal analysis: MSHA, THM, NT and KR. Funding acquisition: THM. Investigation: THM, SR, and ASA. Methodology: MSHA, THM, KR, NT and YHK. Validation: ASA, NHA, NT, and AIA. Visualization: MSHA, THM, and KR. Writing— original draft: MSHA, SR, and THM. Writing— review and editing: KR, NT and YHK. All the authors consented to publish the current draft. All authors contributed to the article and approved the submitted version.
